# Prelacrimal Window Approach in the Management of Odontogenic Maxillary Sinusitis from Dental Foreign Body

**DOI:** 10.1155/2022/1730656

**Published:** 2022-09-12

**Authors:** Saikrishna Ananthapadmanabhan, Anthony Noor, Niranjan Sritharan

**Affiliations:** ^1^Department of Otolaryngology, Nepean Hospital, Kingswood, NSW 2747, Australia; ^2^Department of Otolaryngology, Westmead Hospital, Westmead, NSW 2145, Australia

## Abstract

The floor of the maxillary sinus is partly formed by the alveolar process of the maxilla, and this anatomical relationship forms an interface for collaboration between rhinologists, maxillofacial and dental surgeons, and dentists. Odontogenic maxillary sinusitis (ODMS) occurs secondary to infectious processes of the maxillary molar and premolar teeth or following complications from dental procedures. Extruded dental foreign bodies within the sinus can cause chronic mucosal irritation leading to mucociliary dysfunction and sinogenic symptoms. Anteriorly placed foreign bodies are difficult to access via the conventional endoscopic maxillary antrostomy. Endoscopic approaches to access the anterior maxillary sinus involve extended resection of the medial maxillary wall, potentially with the removal of the inferior turbinate and nasolacrimal duct mobilisation. The prelacrimal window approach (PLWA) is a favourable modification that provides excellent visualisation of the maxillary sinus with minimal tissue resection and displacement. We describe the case of an extruded distobuccal 27 tooth root into the anterior maxillary sinus, presenting with acute sinusitis. The patient was successfully managed via a PLWA. This case represents the importance of recognition of ODMS with early referral to otorhinolaryngologists.

## 1. Introduction

Odontogenic maxillary sinusitis (ODMS) is a common form of paranasal sinus disease and can be secondary to endodontic or periodontal pathology or as a complication of dental procedures, which includes extraction-related oroantral communication or extrusion of dental foreign bodies [1–4]. In the former, there is inflammatory spread through the alveolar process of the maxilla with subsequent infection of the Schneiderian membrane that lines the maxillary sinus. In the latter, the sinus mucosa is vulnerable to an altered microbiome, with oral pathogens and extruded foreign bodies acting as vectors for infection. Ultimately, there is mucosal inflammation, impaired mucociliary clearance and host defences, obstruction of natural sinus drainage pathways, and superimposed bacterial infection.

Foreign bodies in the maxillary sinus are often an iatrogenic complication of dental surgery [5], and may include tooth roots [6], burs [7], implants [8], needles [9], endodontic obturation materials [10, 11], and amalgam [12]. Inadvertent formation of an oroantral communication is the primary route of access for an extruded material, with less common pathways including the tooth socket, the pulp chamber, or operations involving the maxillary antrum. In the case of ODMS from a retained foreign body, the mainstay of surgical treatment is the removal of the offending agent [4]. We describe a case of accidental extrusion of a tooth root into the anterior maxillary sinus, which was removed using an innovative endoscopic technique termed prelacrimal window approach (PLWA).

**Figure 1:**
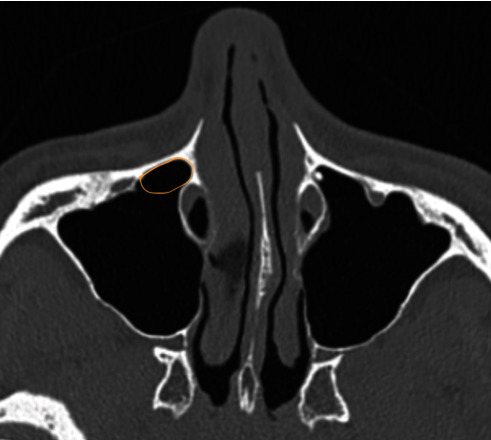
CT representation of the prelacrimal recess shown bounded by the anterior and medial walls of the maxillary sinus, nasolacrimal duct, and infraorbital nerve. The medial boundary defined as the bone between the pyriform aperture and nasolacrimal duct can be instrumented to provide surgical access to the anterior and inferior walls of the maxillary sinus.

The anterior wall of the maxillary sinus has proved to be challenging for surgical access, with difficulty in visualizing and instrumenting this region via the natural ostium [13]. Historical external approaches have included Caldwell-Luc, canine fossa puncture, and midfacial gloving, which have unfavourable morbidity. Subsequent endoscopic endonasal techniques have included Denker's approach and the endoscopic medial maxillectomy (EMM), which usually involve resection of the inferior turbinate (IT) and potentially the nasolacrimal duct (NLD). This may create issues with epiphora and inefficient nasal airflow and humidification causing crusting [14]. The PLWA is a favourable minimal access approach, with preservation of the IT and NLD. The prelacrimal recess, shown in Figure 1, is the area bounded by the medial wall of the maxillary sinus medially, the anterior wall of the maxillary sinus anteriorly, the infraorbital laterally, and the level of the nasolacrimal duct posteriorly. It extends in a craniocaudal direction from the orbital floor to the floor of the nasal cavity. The medial boundary of the prelacrimal recess on the medial wall of the maxillary sinus is defined between the nasolacrimal duct posteriorly and the pyriform aperture anteriorly. The prelacrimal window provides wide surgical access to the maxillary sinus with preservation of normal structures. The technique was first reported by Zhou et al. [15] and has been extrapolated in approaches to the pterygopalatine and infratemporal fossa [16]. Studies have reported the efficacy of the PLWA in addressing multiple maxillary sinus pathologies including inflammatory and neoplastic diseases [17–19]. The dimensions of the prelacrimal window have been described by Simmen et al. [20] and Kashlan and Craig [21].

## 2. Case Description

**Figure 2:**
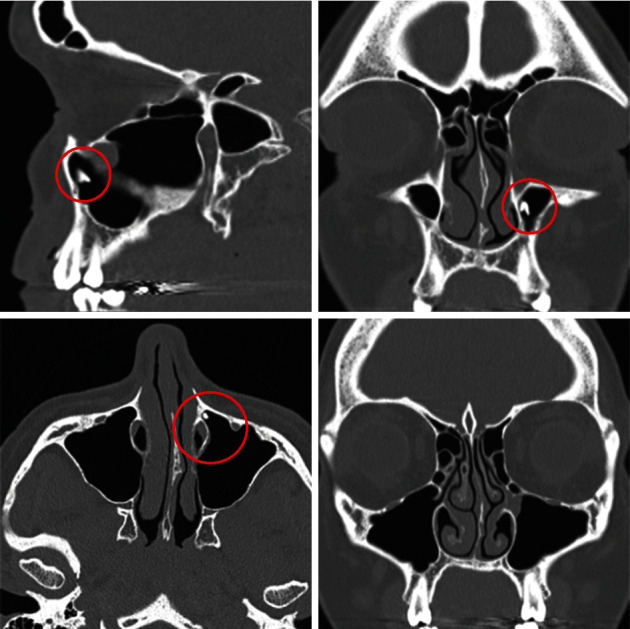
Non-contrast CT paranasal sinuses, bony windows: Top left (sagittal), Top right (coronal), Bottom left (axial) demonstrating 4 × 2 × 5 mm sclerotic, radio-opaque density anteriorly in the left maxillary sinus. The NLD and infraorbital foramen are noted within the red circle. The bottom right (coronal) figure shows lobulated mucosal thickening surrounding the left ostiomeatal unit with occlusion. No periapical lucencies or other radiologic stigmata of endodontic or periodontal disease were present. No osteitis or significant maxillary sinus opacification was observed as the CT was performed within 5 days of the initial dental procedure, and it was organised by the dental team to confirm the existence of the foreign body when the patient had begun to develop headaches but before reporting nasal discharge and infective symptoms. As there was no concern for complicated sinusitis and operative anatomy was already detailed in the initial CT, a repeat CT was not indicated when the patient's nasal obstruction and sinofacial pain were worsening.

A 30-year-old female was referred to our otolaryngology outpatient clinic by the dental surgeons with distobuccal 27 tooth root extrusion into the left maxillary sinus following an extraction 2 weeks previously. This is on a background of known dental caries with previous extractions of teeth 18, 28, 38, and 48. She presented with a 10-day history of left-sided sinofacial pain and progressively worsening headache, with 3 days of anterior and posterior purulent rhinorrhoea. Initial nasal endoscopy at 2 weeks showed pus in the middle meatus. The postnasal space was normal and oral examination revealed a healed 27 socket without evidence of oroantral communication. CT paranasal sinuses with fine slices, arranged by the dental team on day 5 postdental extraction, once headache started to develop, demonstrated a 4 × 2 × 5 m sclerotic density placed anteromedially in the left maxillary sinus with mucosal thickening obstructing the ostiomeatal unit (Figure 2). Her acute infective symptoms were managed with oral antibiotics, nasal irrigation washes, and nasal sprays. The patient reported persistent sinofacial pain and headaches that interfered with her quality of life over the next 2 months, and to provide symptomatic relief and to prevent recurrent exacerbations of acute infective sinusitis, definitive surgical management was pursued. Using the technique described by Simmen et al. [20], a Type II bony prelacrimal recess of 5.6 mm was measured representing suitability for the PLWA.

**Figure 3:**
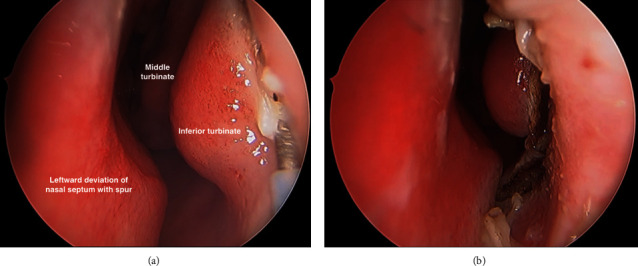
Endoscopic view of nasal cavity. (a) Monopolar diathermy is used to create mucosal incision anterior to the head of the IT; and (b) mucosal incision is extended to the floor of the nasal cavity, allowing a mucoperiosteal flap to be raised.

**Figure 4:**
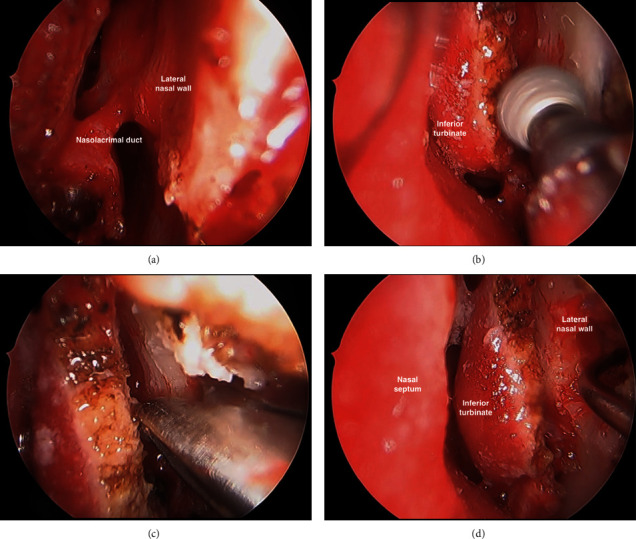
(a) Skeletonization of the NLD. (b) Burr used to commence the prelacrimal medial maxillectomy anterior to the NLD and IT. (c) Bony removal of the lateral nasal wall to access the maxillary sinus. (d) Instrumentation through the prelacrimal window into the maxillary sinus.

**Figure 5:**
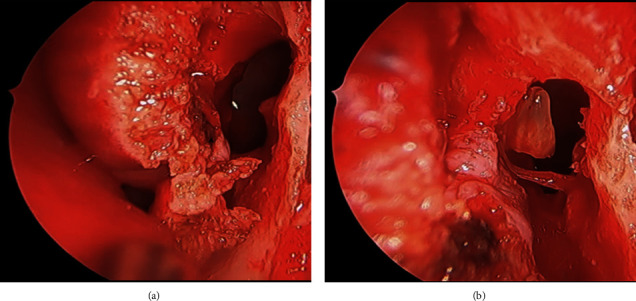
(a) Endoscopic view of the prelacrimal maxillary window accessing the anterior maxillary sinus. (b) Identification and retrieval of the displaced tooth root through the PLWA.

She underwent the prelacrimal maxillary window approach with removal of the dental foreign body and preservation of the IT and NLD (Figures 3–5). The nasal cavity was prepared with cophenylcaine, 1 :  10,000 adrenaline, and topical vasoconstrictors applied onto neuropatties. A mucosal incision was made on the lateral nasal wall anterior to the IT using monopolar diathermy and the mucoperiosteal flap was raised. A burr was used to skeletonise the nasolacrimal apparatus, which was preserved. An endoscopic prelacrimal medial maxillotomy was created to access the anterior part of the maxillary sinus. Excellent visualisation was achieved with the 0° endoscope, and the tooth fragment was identified and removed intact. The flap was replaced and closed with Monocryl sutures. The patient had a good recovery with full resolution of sinus symptoms.

## 3. Discussion

The maxillary sinus is the most common site of paranasal sinus disease. Certain factors that may contribute to this observation include its large volume, the anatomic relationship to the maxillary teeth, and the upward mucociliary drainage pattern towards the superiorly placed natural sinus ostium [22, 23]. Studies have reported that 25–40% of chronic maxillary sinusitis is odontogenic [24] and a 2010 meta-analysis of ODMS suggested that 55% of cases are iatrogenic [25]. Dental instrumentation of the maxillary molars presents the greatest risk to iatrogenic ODMS due to the challenging root canal anatomy and location [4]. Thin or dehiscent bone in the maxillary sinus floor can cause premolar and molar teeth roots to project into the sinus, in close contact with the respiratory epithelium [26]. Previous case reports have documented extrusion of various dental foreign bodies including displaced teeth fragments, endodontic obturation materials, dental burs, drill bits, and amalgam, whereas our case developed symptoms within 2 months of dental surgery, and a latency period between initial insult and sinogenic symptoms of up to 4 years has been described [27]. The management of ODMS includes dental and/or sinus surgery, depending on the underlying aetiology [28, 29]. In cases of foreign body ODMS, removal of the agent is needed to prevent chronic irritation of the Schneiderian membrane and mucociliary dysfunction. The caveat is in the case of implant-related ODMS where removal may introduce the risk of oroantral communication, difficult reimplantation, and cost to the patient [4]. In this cohort, consideration of functional endoscopic sinus surgery (ESS) alone can be entertained to enlarge drainage pathways. The presence of obstruction at the maxillary sinus ostium or ostiomeatal complex and retained dental foreign bodies are strong predictors of the eventual need for ESS [2]. A pressing concern depending on the immune status of the patient and type of foreign body is fungal maxillary sinusitis.

Studies have shown that despite the use of a combination of angled microdebrider blades and antrostomy sites, the anterior and inferior walls of the maxillary sinus remain difficult to access and necessitate extended approaches beyond a standard middle meatal antrostomy [13]. Endoscopic sinus surgery has largely replaced historical nonendoscopic approaches in addressing maxillary sinus disease due to concerns about high rates of permanent facial paraesthesia and neuralgia. With more extensive endonasal approaches, the risk of epiphora, nasal crusting, and ineffective nasal humidification arises. Some authors have raised concerns about the empty nose syndrome with IT resection [30], though this remains controversial. In our patient with ODMS from a dental foreign body, the goal of surgery is straightforward, as the removal of the offending agent should halt sinogenic symptoms. Hence, a minimal access approach is ideal. The IT and medial maxillary wall were replaced to their original anatomical status at the end of the surgery, reducing any potential functional morbidity. A maxillary antrostomy for long-term ventilation and topical medical delivery of the sinus may be considered by some surgeons but was not indicated since the mucosal inflammation causing ostiomeatal complex obstruction will resolve with the removal of the tooth root.

Preoperative CT evaluation of the distance between the anterior maxillary wall and the anterior margin of the NLD is important before PLWA [20]. A radiologic study by Kashlan and Craig [21] provided a comprehensive description of the anteroposterior and superoinferior distances of the prelacrimal recess, with the former distance on the medial wall requiring instrumentation to access the maxillary sinus [21]. The anteroposterior dimension increases in a craniocaudal direction moving from the orbital floor to the opening of the NLD orifice towards the nasal floor, which is consistent with known observations that the nasolacrimal duct has a slight posterior course as it descends down the lateral nasal wall. Distances of the prelacrimal window ≤3 mm can represent high complexity with the need for bone removal and mobilisation of the NLD. Distances ≥7 mm permit the easiest access to the anterior maxillary sinus, and distances between 3 and 7 mm represent intermediate complexity. Even with short distances, modifications to the technique involving the removal of the bony nasolacrimal canal and mobilisation of the NLD to create an adequate working channel have been described in the literature [20, 21, 31]. There is variability among the population in the length of this bony window, with gender differences reported in some studies, with females having shorter distances and greater surgical complexity in accessing the anterior maxillary sinus [32].

## 4. Conclusion

Odontogenic maxillary sinusitis from tooth root extrusion is an iatrogenic complication that should be recognised and referred early. The PLWA is an innovative rhinologic technique that provided excellent surgical access to the anterior maxillary sinus and demonstrated advantages over conventional external and endoscopic techniques.

## Abbreviations

EMM: Endoscopic medial maxillectomy
IT: Inferior turbinate
NLD: Nasolacrimal duct
ODMS: Odontogenic maxillary sinusitis
PLWA: Prelacrimal window approach.
